# Determining the Distinguishing Features of Different Eating Action Types in Germany Using a Mixed-Method Approach

**DOI:** 10.3389/fnut.2021.720392

**Published:** 2021-09-14

**Authors:** Lyn Lampmann, Agnes Emberger-Klein, Klaus Menrad

**Affiliations:** Chair of Marketing and Management of Biogenic Resources, Weihenstephan-Triesdorf University of Applied Sciences, Technical University of Munich Straubing for Biotechnology and Sustainability, Straubing, Germany

**Keywords:** eating action type, data transformation, quantitative content analysis, cross-over analysis, mixed-methods

## Abstract

Food-related behavior is a very complex topic, as it affects the most diverse areas of life. Accordingly, wide varieties of disciplines have already dealt with the topic to understand it better. The result is that there is neither a uniform nutrition knowledge nor a uniform nutrition behavior. In order to reduce the complexity of a field of study, there is the methodical means of type-building. Both commercial and academic studies have already formed nutrition types, either by means of standardized questionnaires or with a specific content focus. However, since both individual and social aspects influence food-related behavior, we investigate how people integrate eating into their everyday life against the background of (competing) individual and social demands by focusing on the individual point of view, for which a mixed methods approach is used. Based on 42 semi-structured, problem-centered interviews conducted in Germany in 2017, we built qualitative food-related types in a first step, which are analyzed in this article using a quantitative content analysis and cross-over analysis to identify the particular distinguishing feature(s) of each type and test them for significance. The results show the prominent characteristics for each type and indicate furthermore that subjectivization, self-determination, the body as an instrument of power, adaptation to the environment and being overstrained with the own behavior are particularly prominent when it comes to eating. Moreover, we clearly identified *The Overstrained* and *The Relaxed* as independent *eating action types*, which we could not find anywhere else. The study shows that interventions, especially for *The Overstrained* and *The Controlled*, are necessary to achieve a relaxed approach to eating in everyday life. At the same time, systematic approaches should be used to intervene in cases of overstraining or controlled behavior.

## Introduction

“Food is so important, and permeates human life in so many ways, that it engages and interacts with almost all of our activities: leisure, the art, sex, work…” (1). The fact that so many areas of daily life can be influenced by food-related behavior has led to different nutritional approaches and views, with neither uniform nor coherent nutritional knowledge nor behavior (2, 3), making it a very complex issue (4–6). Relevant nutritional approaches include food choice approaches [e.g., (7–9)], practice theory approaches [e.g., (6, 10)], psychological approaches [e.g., (11, 12)] and approaches that look at pure physiological food intake [e.g., (13)].

In order to reduce the complexity of a field of study, types can be built. In this process, subjects or objects of an area of study are classified by means of grouping processes based on previously defined characteristics (14). Thus, type-building (15–17) sorts facts according to comparable characteristics (such as e.g., attitudes toward organic food, sports behavior, or the behavior of individuals in group work, etc.) (18). The aim of type-building is to reduce the diversity of data to a few workable and meaningful terms (15) and thus reduce the complexity of situational or personal constructs (19). This approach can be implemented using qualitative methods as well as quantitative methods. In quantitative research types can be built, e.g., *via* cluster analysis procedures (15), and in qualitative research, e.g., *via* type-building methods according to Kuckartz (20).

In Germany, many of the existing nutrition types have been developed by commercial market research institutes (21–25) mainly to explain food demand of differing consumer groups. Generally, these nutrition types are rather heterogenic and the applied methods how they have been built often do not lay open. Nonetheless, these institutes normally apply multivariate statistical methods that examine the consumers regarding their differences or similarities (26). One well-known example are the seven Nestlé nutrition types (25). Based on the nutritional attitudes of the respondents, Nestlé found *The Passionless Pragmatists, The Problem Conscious, The Carefree Full Eaters, The Hunted, The Health Idealists, The Nest Warmer* and *The Modern Multi-Optional* (25). The results showed that the nutrition culture is becoming more and more heterogeneous, but many people are basically pursuing a specific goal with their diet: Fitness, health, personal well-being, self-optimisation or personal appearance (27). In 2012, the Rheingold-Institute has come up with 10 nutrition types. Amongst the results were types called *Mr. and Mrs. Right, Salad Singles, Machines* or *Food Poser* (22). The built types are each associated with a lifestyle, assuming that culinary preferences reflect the lifestyle of the consumer (22). The GfK built eight cooking types, based on the purchasing behavior of the households. The built types have names like *The High-Class Cook, The Everyday Cook, The Weekend Cook, The Warmer* and *The Out-Of-Home Eater* (23). This study provided insight into the changes taking place in the kitchen due to changing role models and the changing working day, assuming furthermore that the development of cooking and eating at home changes the demand for food (23). Additionally, Nielsen Holdings plc published in 2017 seven nutrition types built by requesting 10.000 subjects about their dietary habits, the underlying reasons and the belonging trigger (21). However, one learns very little about the study itself and its purpose.

In addition to the nutrition types of the commercial market research institutes, such nutrition types also were built in Germany in several scientific studies (28–31). With the aim of investigating whether cross-national food consumers can be found, Brunsø et al. (29) developed the food-related lifestyle and defined it as a system of cognitive categories, which were related to different dimensions of nutrition. Based on a survey instrument consisting of 23 sub-dimensions focusing on purchasing behavior, cooking methods, quality aspects, consumption situations and purchasing motives they found five consumer groups in Germany: *The Uninvolved, The Careless, The Conservative, The Rational*, and *The Adventurous* (29). The results suggest a relatively strong trend toward cross-national segments. Nevertheless, different strategies need to be developed for the different target groups to target them with products and get them to buy (29).

Bruhn (28) developed a Lifestyle Decision Typology of Nutritional Behavior resulting in four nutrition types (e.g., *The Disinterested Consumer, The Traditional Consumer* etc.). By applying the food-related lifestyle (FRL), a subsequent cluster analysis and a survey of the dietary behavior, the respondents were segmented according to their lifestyle for food and its significance for health-conscious eating habits (28). The aim was to contribute to behavioral prevention measures in the context of a growing obesity rate. Some possibilities for intervention are pointed out. For example, in addition to promoting health-conscious choices through nutrition education and information, a supportive environment should be created, for example through legislative initiatives, as this is considered the best way to change behavior in the long term (28).

Kluß (30) analyzed the meaning of pleasure, the ability to enjoy and the enjoyment orientation and found e.g., types named: *The Pleasure-Oriented* or *the Rational*. The study was based on a qualitative study and evaluated secondary analytical 26 guideline-based interviews. The interviews dealt with attitudes toward nutrition, eating habits and situations analysis (30). Against the background of the heterogeneity of young people living in an affluent society, this study has shown that education as a resource can be an important factor in consciously dealing with the own eating pleasure and learning how to deal with it (30). In contrast, Stieß and Hayn (31) focused on the nutritional action in everyday life of the respondents to develop strategies that promote a sustainable nutrition. The aim was to illustrate action options and latitudes of the consumers in the context of environment, nutrition and health and their claims toward a sustainable and healthy nutrition. Seven nutrition styles were built, such as *The Disinterested Fast-Fooder* or *The Cheap and Meat Eaters*, based on the purchasing, cooking or comprehensive nutrition orientation and sociodemographic information. The analysis was based on a two-stage empirical survey that started with a qualitative phase and was followed by a quantitative survey (31). The results showed that the change in everyday nutrition leads to a variety of different patterns of action, for example by shifting the main meal to the evening or having a big breakfast together on Sunday (31).

Summarizing, the presented academic typologies focused on a specific topic within food-related behavior such as e.g., sustainability, pleasure or obesity while the commercial studies mainly aimed to explain food demand or nutrition behavior of differing consumer groups. Such insights can be used for commercial as well as policy-driven activities. The first group includes developing differing food products for heterogeneous consumer groups, adapting business and marketing strategies to differing needs of consumers or elaborating adapted information and communication strategies for such purposes. Policy-driven activities that use the insights of typology or segmentation studies include e.g., regulatory initiatives but also information, education or communication campaigns to support e.g., a more healthy food choice in particular of vulnerable groups of the population.

In contrast to the aforementioned commercial as well as academic food-related behavior typologies, we pay special attention to food-related behavior from the individual's point of view and aiming at considering the multiple aspects of how people integrate eating into their everyday lives, while engaging with themselves and their environment (32, 33). The focus is therefore clearly on the individual and his or her challenge to develop the own personality in everyday life and to socialize at the same time (32, 33). Since eating is a very everyday phenomenon that is carried out several times a day alone or with others (12), it is understood as part of this engagement with oneself and the environment, to adapt or to distinguish oneself (11).

For this purpose, the idea of qualitative social research fits best, since it sheds light on the subject and on the in-depth understanding of its actions (34, 35). The application of qualitative interviews was thus ideally suited for the purpose to build a typology.

However, as both research methods, qualitative and quantitative, have their justification and their individual advantages, which mutually benefit each other (36, 37), we decided to apply a mixed methods design for the analysis of the previously built types. Using mixed methods involves collecting and integrating both qualitative and quantitative data to enable analysis of the strengths of both approaches (36).

Therefore, as a first step, we focused in a previous study on how people integrated eating into their everyday lives while engaging with themselves and the environment, thus living out personality development and related socialization (33). The aim was to build types using type-building qualitative content analysis (20) and following Spiekermann's (38) definition of eating action [“Esshandeln,” (38)], which implies action as well as interpretations processes of self-thinking individuals. This enabled us to build seven *eating action types*.

In this article, following the first step, we apply quantitative research methods as a mixed-methods approach to the analysis of the seven *eating action types* using cross over analysis and a quantitative content analysis of communication. We aim to gain breadth and depth in understanding and corroboration (39) of the different types. Thus, the unique feature(s) of each type shall be determined and a better understanding of the various food-related behaviors shall be created. By means of statistical analysis procedures, the special characteristics of the respective types can also tested for their significance. This allows an even deeper insight into those aspects that play a special role for and in the integration of eating into everyday life by emphasizing the differences in food-related behavior of German consumers. Thus, the target of this study is to analyze how people integrate eating into their everyday lives, while engaging with themselves and their environment in Germany, using a mixed-methods approach.

This article is a first step toward generalizing the results of the qualitatively built *eating action type*, using a mixed-method approach that widens the methodological instrumental set in this field of food-related behavior. It can be understood as a “precursor” to the derivation of a general typology that does not have a thematic focus but refers to the population as a whole by focusing on the integration of eating into everyday life and the challenges, their effects and ways of dealing with them from the perspective of the individual.

The results of this study are valuable for nutrition consultancies, food companies, politicians and advisers in order to consider people's differing ways how to integrate eating in their daily lives in their specific activities. This might improve business success of commercial companies, but also enhance effectiveness of political initiatives and advice and education activities in the field of nutrition and eating.

## Methodology

### Design Overview

In 2017, we conducted 42 semi-structured, problem-centered interviews in Bavaria, Germany. Interviews were conducted at the (Self-identifying Institution) in the city of (Self-identifying city), at the Institute for (Self-identifying Institution) in (Self-identifying city) or at the respondents' homes either in the city of (Self-identifying city) or in the city of (Self-identifying city). Participants were contacted via a university email distribution list, a recruitment call in local newspapers or via a list of participants from previous studies.

The interviews focused on how people integrate eating in their everyday lives from their personal point of view (40) and by that living out personality development and related socialization (32, 33).

The interview guideline was developed after reviewing relevant literature on food-related types. It consisted of six sections, two of which are not relevant to this article: 1. the introduction including informed consent, 3. a section on food-related behavior including a 24-h recall and a problem-centered interview about the previous eating day and the last Sunday, 5. a short questionnaire on socio-demographic characteristics, and 6. a conclusion. The interview guideline was pre-tested twice and slightly adapted. All interviews were tape-recorded except for one where notes were taken (35, 41).

The questions of the problem-centered interview took into account the two dimensions of the eating action approach, the action as well as interpretation processes, the extent of which was analyzed for each type (40). These were questions about the food consumed, purchasing and cooking behavior, the design of eating situations, and snacking behavior to capture the action processes. Questions about the importance that eating takes in everyday life, the emotions associated with eating, the importance of eating in society and about inner conflicts regarding eating covered the interpretation processes.

The interviews were tape-recorded and transcribed verbatim. However, all data were anonymised to ensure that participants were not identifiable by the information they gave.

In addition to the interviews, we collected quantitative data using a standardized questionnaire, in which participants were asked to state their gender, age and weight.

All participants gave informed written consent and their participation was voluntary. Ethical approval was not required because we did not collect any sensitive personal data from the participants and did not conduct a medical study. The data protection officer (of self-identifying institution) approved the privacy statement and the documentation of the procedure. We adhered to the established and recorded ethical principles of the Declaration of Helsinki, the German Research Foundation (DFG) and the German Sociological Association (DGS) throughout the study to ensure appropriate conduct.

### Description of Sampling

The aim of the sampling was to obtain a relatively heterogeneous group in terms of the participants' age and gender. Initially, the only criterion for inclusion in the study was a minimum age of 18 years, as parental consent would have been required for younger participants. However, only 37 of the 42 interviews were selected for the analysis. The others were rejected due to two incomplete worksheets, one case of unsuitable responses, one different nationality and one case of an eating disorder. For reasons of comparability, after conducting the interviews we decided not to consider the last two cases for the study either. Nevertheless, saturation was achieved, as no new subjects were identified in the interviews, as confirmed by the data analysis (42). None of those invited to take part refused to participate in the interview. All participants were paid €15 for their assistance. The age range of the respondents was between 18 and 83 years. The study was carried out with 19 men and 18 women. Twenty-six people were of normal weight, eight were pre-obese and three were obese (43). All participants were born in Germany and had been socialized in Western European culture. Three participants were vegans (all female), another three were vegetarians (one male, two female) and the remaining 31 were omnivores. For the sociodemographic characteristics, please see Table 1.

**Table 1:** Overview of the contents of the seven *eating action types* (40).

**Eating action type**	**Short descreption of eating action**	**Essential quote**	**Possible explanation**
Eating as a way of life (*n =* 3)	Characterized by a uniform food concept, such as veganism or a diet based mainly on (wild) herbs. These concepts steer one's own nutritional behavior in a certain direction, which is determined by self-imposed rules and demands out of conviction and which have an impact on the general lifestyle; however, the chosen concept remains entirely endorsed.	*So the last few years I have been VERY busy with it, but I am now putting this knowledge into practice. That's why I don't read so much in books anymore, because I have the knowledge now and integrate it into my everyday life or shopping*.	Spiritualising food and the body through alternative eating. Thus, victory of the mind over the body (11).
The Relaxed (*n =* 7)	Characterized by a conscious and relaxed relationship with their own food-related behavior and the ideas implied. The implementation of the ideas works without effort. Food is strongly associated with positive emotions such as pleasure, joy and anticipation.	*I feel good and comfortable with [the meal], firstly because I know that I enjoyed it, secondly because I had time, that I could enjoy it, that I wasn't under any time pressure and thirdly because I believe that I also fed myself well and correctly and varied*.	Dijker (46) explains the ability of a moderate eating style, which is characterized by perception, consciousness and motivation, representing elements that can all be found in this type.
Eating as a way of self-determination (*N =* 7)	Nutrition is given a high priority, as it is understood as a means of implementing and satisfying one's own needs. The self is at the center of attention and the own needs and demands receive special attention. The need to be free in one's own decisions is high. However, this type does not follow a unified concept such as veganism, but develops its own food concepts, which in particular include the demand for healthy eating.	*But I still always look forward to my salad for lunch. [...] especially because I know that it is homemade and because I know what's in it. Well, that is also important for me. I feel much better when I eat my salad than a meatloaf bread roll I bought at work*.	In self-determination theory (SDT), action can be taken according to autonomous motivation (47, 48), whereby food-related behavior is understood as an opportunity for self-determination and the associated well-being (47, 48).
Eating as a necessary evil (*N =* 3)	Food and everything that belongs to it is of little importance. It is rather understood as something necessary for life. Therefore, little thought is given to food, eating and the behavior associated with it.	*So [eating] is necessary for the preservation of life, but there is no fun in it. I cannot say*.	Age plays an important role here, as age brings with it an increased risk of a lack of social interaction, which is particularly evident during meals (49). This can lead to loneliness. For this reason, these people attach particular importance to their remaining social relationships and rate food as secondary.
The Adaptive (*N =* 5)	Adaptation to others is the characteristic feature of this type. Therefore, the food-related behavior is not implemented independently. Rather, they wait for others to become active in terms of food and they just have to join in. Accordingly, eating together with other (close) people is of special attention.	*Okay, and that's where I adapt. […] on weekends […] the only son who still lives in [.] comes with his wife and one of my grandchildren […] and they bring, I pay, but they bring the food. Therefore, they determine […]*.	According to Chernyakova (50), the preservation of identity in the course of adaptation, the perception of adaptation as a desired action and the existence of appropriate circumstances that enable the subject to make the necessary changes are essential aspects of social and successful adaptation. Accordingly, adaptation reflects behavior that goes along with well-being while preserving one's own identity.
The Overstrained (*N =* 8)	Characterized by overstraining with food. Overstraining results either from personal overload or from external circumstances such as unemployment or illness. Individuals debate with themselves what and how to do things properly, but do not reach a good solution. This leads to behavior patterns, which they do not feel comfortable with and which further unsettle them.	*And sometimes it's stress because when I can't decide what I want, it stresses me. Especially before shopping, because I always think about what I have to buy or what I want to buy and that's a mixture of I'm actually happy that I can buy everything I want, because I don't have anything at home and on the other hand it's like: buy the RIGHT one too*.	It can be assumed that the overstraining is influenced by a discrepancy between implicit and explicit motives. Job et al. (51) showed that motivational discrepancy is related to emotional distress, while emotional distress is (partly) responsible for the connection between motivational discrepancy and food-related behavior. People with motivational discrepancy eat more and prefer unhealthy, tasty food because they want to downregulate the emotional stress caused by the motivational discrepancy (51). *The Overstrained* repeatedly emphasized the discrepancy between internal values, ideas and wishes and external circumstances and demands.
The Controlled (*N =* 4)	Characteristic for this type is the compulsion to keep control over one's own body. This strong need is seen as a unique and particularly characteristic feature of this type, as it determines all food-related behavior.	*But even if I'm hungry, I still don't eat. Because I always pay attention to my kilos. But I actually like doing it [eating] very much*.	Sociological theories of action assume that the body is a controllable instrument that is subject to the will of man (52, 53). Body modifications are associated with success, because athletic bodies represent positively connoted social norms such as performance, endurance and strength (52).

### Data Analysis: Qualitative Content Analysis

The first author was the key person for coding and analysis of the collected information.

To analyze the qualitative interviews, we employed content-structuring qualitative analysis as described by Kuckartz (20) as a first analytical step. This form of analysis aims to identify topics and sub-topics and their systematization and conceptualization. We have worked with the deductive-inductive approach (20), whereby following the guideline, existing (deductive) as well as new (inductive) thematic (close to the material) and analytical (conceptualizing, abstracting) categories were generated and identified (20). Then we wrote case summaries for each interview. In doing so, we worked fact-oriented and close to the text from the perspective of the research question (20).

Afterwards, we performed type-building content analysis (20). This procedure describes a methodological controlled analysis, at which a typology is the result of a grouping process, where a certain social condition is divided into types by means of one or more features. This form of analysis method is particularly useful when qualitative data is available. Moreover, the procedure is described in a comprehensible way and does not fail due to a too high level of abstraction. As a result, the elements of one type are as similar as possible (internal homogeneity), but as different as possible compared to other types (external heterogeneity) (20, 44). To build the typology, we worked as a team. For this purpose, the case summaries were distributed fairly among five independent researchers, who in turn had to summarize the case studies in key words according to the two dimensions of the eating action approach. In two team meetings, the group members presented their cases, in terms of the important features and pinned them on a pin board—either next to or away from the already presented, similar or different cases. The group constantly discussed the results. Through this process, certain groups emerged (20). The result was a rough structure of a typology. The next step was to free the resulting types from their assigned cases. Subsequently, three persons from the teamwork, including the first and second authors, differentiated the emerged types. Thereafter, the cases were assigned to the types again (45). The aim was to construct polythetic types, i.e., cases belonging to a type are as similar as possible, but do not have to be identical (20). Moreover, the results are real types (45). All types built are constructs from model cases. For this purpose, relevant text segments from different cases were selected according to the criterion of plausibility for the type to be described and combined for the description (20).

As can be seen in Table 2, the following seven *eating action types* are the result of that process: *Eating as a way of life, The Relaxed, Eating as self-determination, Eating as a necessary Evil, The Adaptive, The Overstrained* and *The Controlled*.

**Table 2:** Typology table - sociodemographic characteristics, weight and nutrition of *eating action types*.

**Variable**	**Eating as a way of life (*N =* 3)**	**The Relaxed (*N =* 7)**	**Eating as a way of self-determination (*N =* 7)**	**Eating as a necessary evil (*N =* 3)**	**The Adaptive (*N =* 5)**	**The Overstrained (*N =* 8)**	**The Controlled (*N =* 4)**
Gender: female, number (%)	2 (66.7)	4 (57.1)	5 (71.4)	2 (66.7)	1 (20.0)	2 (25.0)	2 (50.0)
Gender: male, number (%)	1 (33.3)	3 (42.9)	2 (28.6)	1 (33.3)	4 (80.0)	6 (75.0)	2 (50.0)
Age, mean value (standard deviation)	42.3 (18.4)	42.9 (24.0)	38.7 (23.3)	81.7 (1.2)	55.4 (22.4)	53.8 (20.9)	45.8 (14.0)
Obese, number (%)	0	1 (14.3)	0	0	1 (20.0)	1 (12.5)	0
Overweight, number (%)	0	1 (14.3)	1 (14.3)	2 (66.7)	1 (20.0)	2 (25.0)	1 (25.0)
Regular weight, number (%)	3 (100.0)	5 (71.4)	6 (85.7)	1 (33.3)	3 (60.0)	5 (62.5)	3 (75.0)
*N =* Number of individuals of the total sample	3 (8.1%)	7 (18.9%)	7 (18.9%)	3 (8.1%)	5 (13.5%)	8 (21.6%)	4 (10.8%)

### Quantitative Content Analysis and Cross-Over Analysis

For the purpose of this study, we conducted a quantitative content analysis with the interviews in a second step, since it is 'a research technique for the systematic, objective, and quantitative description of the manifest content of communication' (54). Thus, it can be used to describe the surface content of communication (55). The benefit of this approach is that it gives an insight into the factors that influence individuals in the seven *eating action types* without having to enquire directly about personal food-related issues and thus run the risk of receiving answers based on the principle of social desirability (12).

We also analyzed the *eating action types* using cross-over analysis to gain a deeper insight of both the essential similarities and differences (56) between the types in terms of their unique features. We applied cross-over analysis based on the quantification of the qualitative variables resulting from qualitative content analysis. This approach is defined as the procedure in which at least one analysis type of one tradition (qualitative/quantitative data) is applied in order to analyze the data of the other tradition (57). It is therefore an inter-paradigmatic analysis (58). Cross-over analysis is especially useful for analyzing complementarity and elucidating (in-)consistencies (59, 60). It is understood in terms of convergent design, which collects and analyses quantitative and qualitative data at similar points in time and is followed by integrated analysis (36). We focused on data transformation as an analysis strategy for complementarity. Data transformation is the conversion of one type of data into another (61). Interpretability is best enhanced when the methods are implemented simultaneously and interactively within a single study (59). Our analysis mainly collected qualitative data, some of which was converted into quantitative data (“quantitizing”) (62) in order to detect regularities and unique features in the qualitative data that would otherwise not be recognizable or communicable (63). Next, the data was analyzed and integrated. The aim was to corroborate the results (“corroboration”), to obtain complementary information on phenomena (“complementarity”) and to expand the information spectrum (“completeness”) (64, 65).

**Figure 1:**
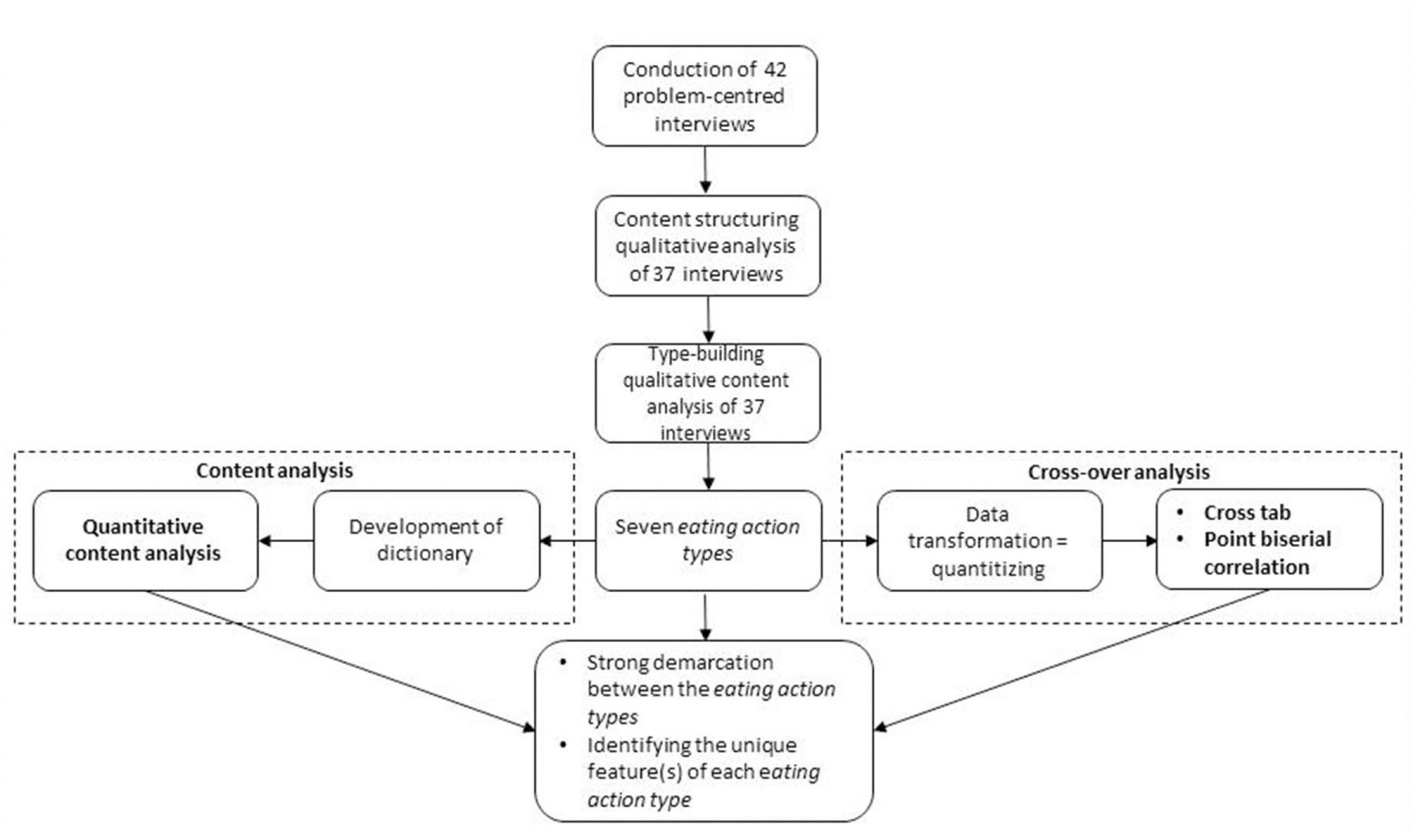
Data collection and study design.

For a detailed overview of the study design applied, please see Figure 1.

### Data Analysis: Mixed Methods

As a basis for our quantitative content analysis, we created a “go list.” For the “go-list,” the MAXQDA (66) software, which included all transcribed interviews, was used to look at the most frequently mentioned words of each *eating action type*. However, only meaningful words were taken into account, i.e., words that have a certain content and a value, such as “pleasant,” “sin,” “indulge,” “gratitude,” or “enjoyment,” and which are not devoid of content, such as the constantly used word “I,” but also “as” or “how,” which are much more frequent in their pure occurrence. In order to ensure that the meaning of the relevant words was related to food, the context was examined in which the word was used. If there was no food reference, this passage was removed manually so that it was not counted as an instance of the word occurring. Thus, the aim of the “go-list” was to identify food-related content and evaluations that were frequently mentioned and thus were particularly characteristic for each type.

Next, we developed a dictionary based on the words included from the “go-list,” using a special function of the MAXQDA software, to create groups of words similar in content and to develop categories by assigning descriptive names to each group. Based on this category system, a quantitative content analysis was performed. In this process, we used the list of references from the respective text passage in the interview and checked whether the statements made by the participants actually reflected the category counted. This procedure was repeated several times until the quantitative content analysis based on the dictionary no longer gave misleading results.

Initially, our dictionary contained 13 categories. However, we excluded four categories, because they contained too few words compared to the other categories. Thus, all categories contain a minimum of seven words. If a word belonging to a certain category was mentioned several times in a single sentence, we only counted that category once.

In addition, we performed a cross tab analysis of the *eating action types* and the categories that evolved in the course of the qualitative content analysis.

We complemented and checked the results of the cross tab by evaluating the point biserial correlation using SPSS (67). The point biserial correlation is particularly suited to the analysis of relationships between an interval-scaled variable (number of counts of the categories) and a dichotomous variable (*eating action type*) (68). We quantified the main categories and subcategories of the qualitative content analysis by transforming them into numeric variables (number of mentions) using MAXQDA.

## Results

This section presents the main findings of the analysis in the form of a single paragraph summary for each *eating action type*.

Table 3 shows the results of the quantitative content analysis for each *eating action type*. The most frequent content is “body reference.” It should be noted, however, that seemingly identical content can have quite different origins and consequences. For example, in *The Relaxed* “body reference” stands for the need to be full and well-fed, whereof in *The Overstrained* it stands for the physical discomfort triggered by the own food-related behavior, which is perceived as not being good. Despite these differences in content, both types emphasize the relevance of body sensations.

**Table 3:** Results of the quantitative content analysis based on categories.

**Dimension**	**Eating as a way of life** **(*N =* 3)**	**The Relaxed** **(*N =* 7)**	**Eating as self-determination** **(*N =* 7)**	**Eating as a necessary evil** **(*N =* 3)**	**The Adaptive** **(*N =* 5)**	**The Overstrained** **(*N =* 8)**	**The Controlled** **(*N =* 4)**
Most frequent content	Body reference (18)^*^	Body reference (16)	Intended behavior (21)	Habits (5)	Community (9)	Body reference (20)	Body reference (20)
Second-most frequent content	Subjectivization (10)	Community (15)	Community (18)		Habits (7)	Intended behavior (17)	Control behavior (16)

* *Number in brackets: counted citations*.

In order to determine the unique features of each *eating action type*, we have listed the important qualitative categories identified for the types in Table 4. However, behavior can vary among individuals of the same type. Thus, the qualitative categories in Table 4 do not necessarily apply to every individual to the same extent. For example, Table 4 shows that for *Eating as a way of life* the behavior change was through information acquisition, which cannot be found for any other *eating action type*. The same is true for *The Adaptive*, where eating is a very important part of family life and is also unique in comparison.

**Table 4:** Cross table for selected categories based on qualitative content analysis: percentage of number *N* = individuals.

**Category**	**Eating as a way of life** **(*N =* 3)**	**The Relaxed** **(*N =* 7)**	**Eating as self-determination** **(*N =* 7)**	**Eating as a necessary evil** **(*N =* 3)**	**The Adaptive** **(*N =* 5)**	**The Overstrained** **(*N =* 8)**	**The Controlled** **(*N =* 4)**
Behavioral change by gathering of information	100.00	0.00	0.00	0.00	0.00	0.00	25.00
Dealing with own demands	66.67	0.00	0.00	0.00	0.00	25.00	0.00
Rewarding	66.67	14.29	14.29	0.00	0.00	25.00	0.00
Relating to others	100.00	0.00	28.57	0.00	0.00	25.00	50.00
Belief in natural physical needs	66.67	14.29	14.29	0.00	0.00	0.00	100.00
Reasoning of own consumption behavior	66.67	28.57	57.14	0.00	0.00	75.00	75.00
View of society	100.00	14.29	42.86	0.00	60.00	50.00	50.00
Conscious food intake	66.67	57.14	42.86	0.00	20.00	37.50	50.00
Good conscience	100.00	57.14	42.86	0.00	20.00	25.00	25.00
Positive affect	33.33	100.00	42.86	66.67	60.00	25.00	50.00
Justification	0.00	57.14	14.29	33.33	20.00	12.50	25.00
Importance of being satisfied	0.00	28.57	0.00	0.00	40.00	0.00	0.00
Self-determined daily structure	0.00	0.00	28.57	0.00	0.00	0.00	0.00
Development of own structures	0.00	0.00	42.86	0.00	20.00	12.50	0.00
Homemade	33.33	28.57	57.14	0.00	20.00	50.00	50.00
Rarely cooks	33.33	0.00	14.29	100.00	20.00	25.00	25.00
Weight control	0.00	28.57	28.57	66.67	40.00	75.00	75.00
Development of own individual health concept	66.67	57.14	42.86	33.33	60.00	50.00	25.00
Adaptation to others	33.33	14.29	14.29	0.00	60.00	12.50	0.00
Important part of family life	0.00	42.86	14.29	0.00	80.00	12.50	0.00
Overstraining	33.33	14.29	28.57	0.00	0.00	62.50	25.00
Negative affect	0.00	0.00	28.57	0.00	0.00	75.00	25.00
Eating as a distraction	0.00	14.29	14.29	0.00	0.00	37.50	0.00
Guilty conscience	33.33	14.29	28.57	0.00	0.00	50.00	25.00
Balancing behavior	0.00	14.29	0.00	0.00	40.00	12.50	100.00
Weighing process	0.00	28.57	28.57	0.00	0.00	25.00	75.00
Exceptions (from control behavior)	0.00	14.29	28.57	0.00	0.00	0.00	50.00

To analyze which qualitative categories are able to withstand statistical calculation and are thus significant and indicative for each type, we have calculated the point biserial correlations for each category and each *eating action type*, which are shown in Table 5. The values highlighted in bold are significant values calculated by the point biserial correlation.

**Table 5:** Point biserial correlation [Significances (2 sided)] (*n* = 37) of the *eating action types* with important categories found in the qualitative content analysis.

**Variable**	**Eating as a way of life** **(*N =* 3)**	**The Relaxed** **(*N =* 7)**	**Eating as self-determination (*N =* 7)**	**Eating as a necessary evil** **(*N =* 3)**	**The Adaptive** **(*N =* 5)**	**The Overstrained** **(*N =* 8)**	**The Controlled** **(*N =* 4)**
Behavioral change by gathering of information	0.905^**^ **(0.000)**	−0.159 (0.349)	−0.159 (0.349)	−0.098 (0.566)	−0.130 (0.444)	−0.172 (0.307)	0.062 (0.716)
Dealing with own demands	0.635^**^ **(0.000)**	−0.132 (0.435)	−0.132 (0.435)	−0.081 (0.632)	−0.108 (0.524)	0.046 (0.786)	−0.095 (0.575)
Rewarding	0.512^**^ **(0.001)**	−0.077 (0.652)	−0.077 (0.652)	−0.114 (0.501)	−0.152 (0.369)	0.109 (0.519)	−0.134 (0.430)
Relating to others	0.442^**^ **(0.006)**	−0.203 (0.228)	−0.090 (0.596)	−0.125 (0.462)	−0.166 (0.326)	−0.113 (0.504)	0.424^**^ **(0.009)**
Belief in natural physical needs	0.713^**^ **(0.000)**	−0.105 (0.538)	−0.105 (0.538)	−0.111 (0.512)	−0.148 (0.381)	−0.197 (0.243)	0.159 (0.346)
Reasoning of own consumption behavior	0.121 (0.477)	−0.162 (0.338)	0.121 (0.476)	−0.204 (0.226)	−0.272 (0.104)	0.285 (0.087)	0.046 (0.786)
View of society	0.257 (0.125)	−0.232 (0.168)	0.126 (0.459)	−0.162 (0.337)	−0.067 (0.692)	0.145 (0.391)	−0.068 (0.691)
Conscious food intake	0.053 (0.755)	0.029 (0.864)	0.029 (0.864)	−0.192 (0.254)	−0.125 (0.460)	0.148 (0.382)	−0.010 (0.955)
Good conscience	0.264 (0.114)	0.105 (0.536)	0.213 (0.205)	−0.201 (0.232)	−0.144 (0.395)	−0.150 (0.375)	−0.099 (0.558)
Positive affect	−0.141 (0.407)	0.565^**^ **(0.000)**	−0.160 (0.346)	0.008 (0.962)	−0.029 (0.865)	−0.216 (0.200)	−0.078 (0.648)
Justification	0.121 (0.477)	−0.162 (0.338)	0.121 (0.476)	−0.204 (0.226)	−0.272 (0.104)	0.285 (0.087)	0.046 (0.786)
Importance of being satisfied	−0.086 (0.612)	0.416^*^ **(0.010)**	−0.140 (0.407)	−0.086 (0.612)	0.098 (0.565)	−0.153 (0.367)	−0.101 (0.551)
Self–determined daily structure	−0.071 (0.676)	−0.115 (0.496)	0.495^**^ **(0.002)**	−0.071 (0.676)	−0.094 (0.578)	−0.126 (0.459)	−0.083 (0.624)
Development of own structures	−0.117 (0.489)	−0.191 (0.258)	0.415^*^ **(0.011)**	−0.117 (0.489)	0.075 (0.659)	−0.016 (0.927)	−0.138 (0.417)
Homemade	0.205 (0.224)	0.032 (0.851)	0.032 (0.851)	−0.194 (0.249)	−0.099 (0.560)	−0.013 (0.941)	0.036 (0.834)
Rarely cooks	−0.028 (0.867)	−0.260 (0.120)	−0.077 (0.651)	0.498^**^ **(0.002)**	−0.003 (0.987)	−0.021 (0.901)	0.044 (0.797)
Weight control	−0.213 (0.205)	0.020 (0.907)	−0.265 (0.113)	0.079 (0.642)	−0.144 (0.396)	0.243 (0.147)	0.264 (0.115)
Development of own individual concept	−0.003 (0.986)	0.180 (0.287)	−0.137 (0.419)	−0.117 (0.492)	0.056 (0.740)	0.045 (0.792)	−0.070 (0.680)
Adaptation to others	0.138 (0.416)	−0.119 (0.483)	−0.025 (0.882)	−0.131 (0.441)	0.470^**^ **(0.003)**	−0.142 (0.402)	−0.153 (0.365)
Important part of family life	−0.148 (0.382)	0.053 (0.756)	−0.143 (0.400)	−0.148 (0.382)	0.587^**^ **(0.000)**	−0.075 (0.657)	−0.173 (0.305)
Overstraining	0.078 (0.647)	−0.182 (0.280)	−0.131 (0.440)	−0.144 (0.396)	−0.191 (0.257)	0.579^**^ **(0.000)**	−0.104 (0.542)
Negative affect	−0.160 (0.344)	−0.260 (0.120)	0.015 (0.930)	−0.160 (0.344)	−0.213 (0.205)	0.634^**^ **(0.000)**	−0.014 (0.934)
Eating as a distraction	−0.101 (0.551)	−0.078 (0.648)	−0.078 (0.648)	−0.101 (0.551)	−0.135 (0.427)	0.483^**^ **(0.002)**	−0.119 (0.485)
Guilty conscience	−0.052 (0.760)	−0.107 (0.529)	0.094 (0.579)	−0.148 (0.382)	−0.197 (0.242)	0.376^*^ **(0.022)**	−0.089 (0.600**)**
Balancing behavior	−0.138 (0.416)	0.072 (0.672)	−0.224 (0.183)	−0.138 (0.416)	0.043 (0.802)	−0.150 (0.377)	0.585^**^ **(0.000)**
Weighing process	−0.147 (0.386)	0.014 (0.936)	−0.070 (0.679)	−0.147 (0.386)	−0.195 (0.247)	0.061 (0.722)	0.464^**^ **(0.004)**
Exceptions (from control behavior)	−0.080 (0.638)	−0.061 (0.719)	0.007 (0.965)	−0.080 (0.638)	−0.106 (0.531)	−0.141 (0.404)	0.513^**^ **(0.001)**

** *The correlation is significant at the level of 0.01 (2-sided)*.

* *The correlation is significant at the level of 0.05 (2-sided)*.

Again, an example of a highly significant correlation is the behavioral change through information acquisition in *Eating as a way of life*. Also highly significant is the overstraining and the negative affect toward the own food-related behavior of *The Overstrained*. Another significant value is the compensatory behavior of *The Controlled*, which is used, for example, by skipping meals in order to compensate for previous meals in their effect on the body.

It is also worth mentioning that five significant values could be calculated from the quantitative content analysis for *Eating as a way of life*, but only one for *Eating as necessary Evil*, i.e., for the group for whom food is simply not of great importance.

Finally, we have combined the results of our study in Table 6. In the following, we will go into more detail about the findings shown in Table 6. Above all, this table shows how the individual *eating action types* are reflected in actual unique features.

**Table 6:** Joint presentation of qualitative and quantitative data.

**Eating action type**	**Distinctive features based on quantitative analysis of qualitative data**
Eating as a way of life	**Main food-related contents:** Body reference; subjectivization **Significant unique features:** Behavioral change by gathering information; Dealing with own demands; rewarding; relating to others; belief in natural physical needs
The relaxed	**Main food-related contents:** Body reference; community **Significant unique features:** Positive affect toward eating day(s); importance of being satisfied
Eating as self-determination	**Main food-related contents:** Intended behavior; community **Significant unique features:** Self-determined daily structure; development of own individual structures
Eating as a necessary evil	**Main food-related content:** Habits **Significant unique features:** Rarely cooks
The adaptive	**Main food-related contents:** Community; habits **Significant unique features:** Adaptation to others; eating as an important part of family life
The overstrained	**Main food-related contents:** Body reference; intended behavior **Significant unique features:** Overstraining; negative affect toward eating day(s); eating as distraction; guilty conscience
The controlled	**Main food-related contents:** Body reference; control behavior **Significant unique features:** Relating to others; balancing behavior; weighing process; exceptions (from control behavior)

### Eating as a Way of Life

This type is represented by two women and one man. All three are of regular weight. It is the *eating action type* with the second-lowest age, with an average of 42.3 years.

*Eating as a way of life* is characterized by a unified and life-influencing nutritional idea. Thus, the lifestyle, which implies an idea of an ideal diet, is oriented toward one's own demands and needs, i.e., living in harmony with nature, the body and the mind. The “body reference” (*n* = 18) therefore illustrates a strong need for body awareness, because the satisfaction of one's own demands and needs and thus the communication of a positive body awareness underlines the perceived positive effects of the idea of an ideal diet. “Subjectivization” (*n* = 10) takes the form of repeatedly relating one's own more radical lifestyle to that of others, along with the consequences and difficulties that go along with this. By gathering information, these people have adapted their behavior toward an idea of an ideal nutrition (*r* = 0.905, *p* < 0.001). The main reason for the behavioral change is the belief in natural physical needs (living in harmony with nature) (*r* = 0.713, *p* < 0.001), which is an integral factor of the diet. The concept enables individuals of this type to reward themselves (*r* = 0.512, *p* < 0.001), because, among other things, they repeatedly have to manage their own claims toward others (*r* = 0.635, *p* < 0.001), which underlines the importance of subjectivization by making constant reference to them (*r* = 0.442, *p* < 0.01).

### The Relaxed

*The Relaxed* is represented by three men and four women, with an average age of 42.9 years. Five are of normal weight, one is overweight and one is obese. *The Relaxed* is characterized by a distinctly casual attitude to food. At the same time, emphasis is also placed on the person's own well-being. The quantitative content analysis identifies “body references” (*n* = 16) as a main topic, revealing that this type considers physical well-being to be important. Thus, *The Relaxed* makes reference to the body to underline the importance of eating a full and healthy diet. The second most frequent content is “community” (*n* = 15). For this type, “community” is important because of the benefits and positive associations of eating with other people and the way their eating behavior and meal requirements are affected in the presence of others. Their unique and decisive feature is their relaxed attitude toward food and their own diet (*r* = 0.565, *p* < 0.001) along with a desire to feel replete after eating (*r* = 0.416, *p* < 0.05).

### Eating as Self-Determination

Two men and five women represent this type. With an average age of 38.7 years, it is the youngest group of *eating action types*. Only one individual of this type is overweight, while the others are of normal weight.

This type is strongly focused on itself in terms of independence from others. The feeling of being dependent is considered stressful by this type. The “intended behavior” (*n* = 21) is one of the issues that *Eating as self-determination* often refers to, which emphasizes the endeavor to use fresh food, to prepare meals according to one's needs and to consume in a moderate, regular and healthy way. The behavior therefore refers to the use, preparation and consumption of food. The intended behavior again underlines the other aspect, the need to be independent of others. *Eating as self-determination* thus refers to “community” (*n* = 18) to underline his or her own distinctiveness from other people. The personal attitude toward food is then clear: the individuals in this type set themselves apart from and compare themselves with others, and the behavior of others is reflected on and evaluated. Since a self-determined daily structure is of central importance (*r* = 0.495. *p* < 0.01), this category is not mentioned by any other type: The examination of society serves to delimit and emphasize the self and its needs, as does the development of one's own structures and the abandoning of behaviors learned from parents (*r* = 0.415, *p* < 0.05).

### Eating as a Necessary Evil

Two women and one man are the representatives of this type, which is the oldest of all the *eating action types* with an average age of 81.7 years. Two of the individuals are overweight and one is of normal weight.

For this type, neither eating itself nor any other food-related behavior plays any relevant role; eating is considered to have no meaning or value. The main finding of the content analysis was found during the interviews to be “habits” (*n* = 5). This underlines the routines that characterize food-related behavior, indicating the insignificance of food and eating. The same established and familiar behaviors shape this type's food-related behavior, indicating an unwillingness to address the topic more intensively. The fact that this type rarely cooks is also fitting (*r* = 0.498, *p* < 0.01).

### The Adaptive

Four men and one woman represent this *eating action type*. The average age of this type is 55.4 years; three are of normal weight, one person is overweight and one is obese.

The *Adaptive* type is mainly characterized by a tendency to adapt to others. This is underlined by the results of the quantitative content analysis, which shows the importance of the community for providing a food-related behavior; “community” (*n* = 9) is therefore the main topic of this type. *The Adaptive* makes reference to “community,” indicating that social interaction plays a significant role. Food and eating is understood as an essential aspect of family and social life (*r* = 0.587, *p* < 0.001). This fact is always in the foreground when it comes to eating and leads to a situational adaptation of the type's own food-related behavior to that of others (*r* = 0.470, *p* < 0.01). A noteworthy aspect is the frequent use of *us* or *we*, which underpins the adaptation to others. A further important issue is “habits” (*n* = 7), which indicate a partly shared standardized behavior. *The Adaptive* refers to this content by emphasizing family meal rituals.

### The Overstrained

Six men and two women, with an average age of 53.8 years, represent *The Overstrained*. Five people of this type have normal weight, two are overweight and one is obese.

*The Overstrained* is characterized by a failure to implement a consistent positive attitude toward food. Thus, this type makes repeated “body references” (*n* = 20) due to the negative impact of their inconsistent and stressful behavior on the body. However, these references differ greatly due to the individual's failure to meet existing demands (e.g., having no physical limitations despite having a high weight, a lack of self-respect for the body, a desire to stay fit, an unwanted feeling of fullness). The “intended behavior” (*n* = 17) stands for all the attempts that are made in the mind to change the situation. Significantly, these attempts tend to remain in the mind, and no actual changes occur. People of this type are aware of what they should (and should not) be doing, but for various reasons, they do not do it, which then leads to overstraining (*r* = 0.579, *p* < 0.001). In general, this leads to a negative affect toward eating day(s) (*r* = 0.634, *p* < 0.001). This in turn leads to using food as a distraction (*r* = 0.483, *p* < 0.01), followed by a guilty conscience (*r* = 0.367, *p* < 0.05), which also results from an inability to change the situation.

### The Controlled

This type is represented by two men and two women. The average age is 45.8 years, and three individuals are of normal weight, while one is overweight.

The *Controlled* type is characterized by a highly controlled behavior. The emphasis is on the body, which is required to meet the person's own high standards. The results of the quantitative content analysis revealed “body reference” (*n* = 20) as the main issue for this type. For *The Controlled*, the all-influencing importance of low body weight is emphasized, leading to generally controlling behavior. Thus, “control behavior” (*n* = 16) stands here for the constant need to meet one's own physical demands. To achieve this, the abandonment of certain foods or meals is an acceptable price (*r* = 0.585, *p* < 0.001). Body image is of particular importance, weakened or strengthened by comparisons with others (*r* = 0.424, *p* < 0.01). This constant preoccupation with one's own body leads to balancing previous and present consumption and weighing up present consumption with what has already been consumed and what may still be consumed (*r* = 0.464, *p* < 0.01). Although this behavior is very restrictive, it is not considered to be an eating disorder, because people of this type repeatedly allow themselves exceptions to the controlled behavior (*r* = 0.513, *p* < 0.001).

## Discussion and Conclusions

The purpose of this paper was to analyze more deeply the previously built *eating action types* by bringing their decisive features more into focus. To do this, we applied quantitative analysis of qualitative data.

Through our analysis, we were able to identify the different challenges, their effects and ways of dealing with them that occur through eating together and eating alone in everyday life, through which personality and, consequently, socialization are lived. The quantification of our data highlights the essential characteristics of the individual types, thus showing the different focal points in dealing with the integration of food into everyday life. These in turn can be clustered and so there are seven ways in how people integrate food into their everyday lives, their reasons and the challenges they face in doing so. Therefore, *Eating as a way of life* defends the own behavior, which is strongly influenced by the belief in natural physical needs, and by constantly establishing and presenting the reference to others in the context of the own demands. The subjective view plays an essential role, encompassing a unified system that is defended and lived by. For *The Relaxed*, positive associations with food play a role above all: the strong need to be sufficiently and well-nourished is pursued with certain lightness. For *Eating as self-determination*, the self-determined daily structure is in the foreground; it focuses on the development of individual structures and rejects the familiar. That eating hardly plays a role for *Eating as a necessary Evil* is confirmed by the rare cooking. The adaptation to others, which stands in strong contrast to *Eating as self-determination*, i.e., not forming own structures, but rather going along with it and focusing on the community aspect, reflects the behavior of *The Adaptive*. The challenges of *The Overstrained* are only met with overstraining, which is reflected in the bad feeling about food-related behavior, the guilty conscience, but also in distraction behavior. *The Controlled* are constantly negotiating and weighing up what is allowed and what is not, in order to feel good in their own bodies. In doing so, they constantly relate to others, compare themselves and devalue the behavior of others.

### Methodological Considerations

Quantitative analysis of the qualitative data enables an enhanced distinction between individual *eating action types* and pinpoints their particular distinguishing features, giving us a detailed insight into the characteristics of the types. This article shows that quantitative analysis techniques can also be used with small case numbers and can contribute to a general understanding of the thematic content. This indicates the benefit of the cross-over analysis as the cross tab stressed special characteristics and the results of the point biserial correlation underline significant unique features of these special characteristics. The result is an increased and more focused differentiation between the *eating action types* that give a clear insight into how people integrate eating into everyday lives while engaging with themselves and the environment.

The limitations of the methods used in this project are due to the small number of cases. Thus, it is not possible to describe this as a representative study. The results of the study should be tested to ascertain their significance for a representative cross section of the population.

It would also be interesting to determine to what extent our approach and the corresponding results can be transferred to other areas of consumption. This would serve to test and verify our design. Additionally, after repeated testing, our approach could serve as the basis for developing an instrument that can identify different types in differing areas of consumption.

Furthermore, the development of a dictionary requires a lot of manual effort, since MAXQDA cannot evaluate content logic. Accordingly, each assignment requires human verification, which is very time-consuming and error-prone. Evaluation by a scientist is a subjective process. On grounds of capacity, only one scientist, the first author, reviewed the text passages assigned to the respective categories. Thus, it cannot be excluded that this also influenced the results of this study.

Since this study was conducted in one particular region of Germany (Bavaria), it is necessary to investigate whether the *eating action types* identified can be verified for the whole area of Germany.

Since food-related behavior differ in different nations and cultures, other questions concerns what changes might occur if the approach were to be transferred to other countries or cultural areas.

### Discussion of Factual Findings

As a result of changes in society and the multiple options that are now available, eating culture has become more heterogeneous in Germany (27, 69). Our study confirms these findings by highlighting the peculiarity of each *eating action type* to determine the unique features of each individual *eating action type*. Nitzko and Spiller (70) revealed that pleasure orientation, slimness and well-being, and health and diversity are essential factors that influence food-related behavior. All of these aspects can be found in our types: pleasure orientation is an important aspect for *The Relaxed*, slimness is of high relevance for *The Controlled*, well-being is significant for every *eating action type*, and health is especially important for *Eating as a way of life*, whereas diversity is represented by the different types.

A detailed comparison of the findings of this study with previous segmentation studies in Germany is restricted by differences in the studies' foci. Our study focuses on the *eating action* question of how people integrate eating into everyday lives, while other studies mainly focus on the food itself, e.g., the question of what is eaten (21–23, 25) or have special thematic foci (28–31). Thus, we highlight findings of this study that are comparable with each other.

With regard to the Nestlé Nutrition Types (25) we find that *Eating as a way of life* is comparable with *The health idealists* [”Gesundheitsidealisten,” (25)] in the sense that they predominantly comprise ecologically conscious women who wish to live in harmony with nature, adapting their current lifestyle by obtaining new information (25). We did not find a comparison group for *The Relaxed*. The Nestlé nutrition study also includes a *Modern Multi-Optional* type [“Die modernen Multi-Optionalen,” (25)] which can be compared with *Eating as self-determination*. Both groups have in common that they are relatively young, with ages of 26–55 and live in a dichotomy between their own high demands and a tight time budget. *The dispassionate pragmatists* [“Die leidenschaftslosen Pragmatiker,” (25)] can be compared with *Eating as a necessary Evil*. Similarities can be found in the age structure, the level of sophistication, and the satisfaction with the status quo. Food intake in this group is simply seen as a means to an end, but there are fixed dietary rituals and emphasis is placed on regular meals. Characteristics such as those that we found in the *Adaptive* type can also be seen in *The Nest Warmers* [“Nestwärmer,” (25)]. This group covers all age groups. Here, family and tradition are very important, harmony is strived for and joint meals with family and friends are very important.

Interestingly, *The Overstrained* is not found as an independent group in any other nutritional typology (21–23, 25, 28–31). Only some types mentioning inner conflicts and ambivalences are identified in previous studies. However, these types do not have a general character, but are concerned with partial aspects (31). However, *The Overstrained* is actually the largest group in our study, with a total of eight persons. Hayn (69) and Jastran et al. (71) refer to the fact that once established, everyday actions are not constantly reflected upon because they reduce complexity, offer strong relief potential and provide stability and security “*because life runs more smoothly when things become predictable and expected from day to day and week to week”* (71). However, these everyday actions have to be actively constructed, stabilized, maintained and changed by each individual (69). With regard to *The Overstrained*, the results of our study suggest that many people fail to implement actively everyday food-related behavior due to internal or external changes that are actively affecting them and that lead to non-well-being due to implicit motives that are not congruent with their external motives (51, 72). However, this motive incongruence cannot be revealed by examining food-related behavior only with a standardized questionnaire. Rather, standardized questionnaires describe how people actually behave; but it is hard to determine by means of closed questions whether behind the behavior described lies a (great) overstraining. Only through the combination of qualitative and quantitative research methods, it was possible, on the one hand, to identify the group of *The Overstrained* qualitatively and, on the other hand, to confirm and emphasize it through statistical analysis. Otherwise, overstraining in the context of food-related behavior is only taken into account by studies focusing on people with specific eating disorders (anorexia, bulimia, etc.) (73). However, as our study suggests, there are people with a healthy mental attitude to food who nevertheless feel overstrained. Moreover, the absolute number of overweight and obese people is highest in *The Overstrained* group. Taking the definition of the WHO, 37.5% of people of *The Overstrained* weigh too much (43). Bruhn (28) shows that social changes and changes in the environment are the main reasons for the rapid increase in overweight and obesity (28). Only *Eating as a necessary Evil* and *The Adaptive* show higher rates of overweight and obesity. However, *Eating as a necessary Evil* is the oldest *eating action type*, and weight gain in old age is due to changes in a person's metabolism and energy requirements (74).

*The Controlled* can best be compared with the *Fitness-oriented and ambitious* type [“Die fitnessorientierten Ambitionierten,” (31)] of the study by Stieß and Hayn (31). This type is characterized, among other things, by an effort to maintain high levels of performance and fitness and to keep the body attractive through controlled eating. Individual success is measured primarily in terms of the perception of others, which is why some people attach so much importance to their own appearance (31).

Another interesting aspect is that of the sense of community created by eating together. Joint meals are of great importance to many people, even though the community-building role of eating is actually becoming less important (27). Our study confirms both findings, as eating together is indeed of great importance to many of our participants, while some struggle with the food-related behavior of other people, placing the focus on their own food-related behavior (*Eating as a way of life, The Relaxed, Eating as self-determination* and *The Controlled*). For *Eating as a necessary Evil*, eating as such does not play a relevant role, therefore nor does eating together.

With this study, we have shown that quantitative analysis of the qualitative data was able to identify an improved distinction between the individual eating action types and show their particular distinguishing features, giving us a detailed insight into the characteristics of each type. As a major result, we did not find a comparison group for *The Relaxed*, nor for *The Overstrained* in previous literature. Interestingly, these two are the groups that seem to be the most opposed to each other that have so far received little attention in terms of their extent as a single group. The result of *The Overstrained* as a distinct group deserves special emphasis. This group does not suffer from a mental eating disorder; rather they would like to know a relaxed way of integrating food into their everyday life. Yet they fail and often suffer from obesity. The desire to find a sensible way to integrate eating into everyday life should be taken into account for people of this type by nutritional counseling, policies and advertisers.

In this study, we were able to show the main content of each type when it comes to eating. It was shown that an idea of an ideal nutrition leading to physically and ethically well-being, having a relaxed attitude toward eating, self-determination, the body as an instrument of control, adaptation and overstraining play relevant roles when it comes to how people integrate eating into their everyday life and thus experience personality development and related socialization. In the group comparison, it became clear that the reference to the body occurs repeatedly, although the reference can have different origins, e.g., the need to feel good physically, or the need to maintain control over the body or to live in harmony with nature. In addition, the reference to the “community” is increasingly established, but here too from different perspectives, either because the presence of others is stressful or relaxing. The need for subjectivization and for control is unique: these two contents can be found in only one type each and thus distinguish them in particular. The cross-over analysis, in turn, confirms the results of the qualitative built types and underpins the respective *eating action types* in their characteristics, with which they distinguish themselves from each other. Thus, this study is the first food-related consumer segmentation study for Germany that uses a mixed-methods approach, which expands the methodological toolkit in this field.

In this study, we have shown what methods are applied individually when it comes to how people integrate eating into their everyday lives and thus experience personality development and related socialization. Special intervention is needed for *The Overstrained*. Therefore, the reasons for the overstrain must be looked at closely, which can also be caused by external factors, so personal contact is needed that takes the social system into account [keyword: systemic approach (75)]. *The Controlled* could also profit from systematic approaches, because of their constantly reproduced reference to their environment and its evaluation. Moreover, for *The Controlled*, education about physical needs would probably be useful, because the constant compensatory behavior is exhausting for the body. It is much more important to have regular mealtimes and a balanced diet. One idea would be to cooperate with the people of *The Relaxed* and benefit from their experiences, and from the ways to establish a relaxed approach to eating, for example by having participatory processes, where mutual reporting and learning from each other is possible. This seems to make sense insofar as classical nutrition communication often fails due to its abstraction and the lack of a realistic assessment to the target groups (76). A participative procedure would be a procedure at eye level, where solutions are worked out together (77). The results of this study can also help to support the need for self-determined daily structure by creating individual plans for *Eating as a self-determination* with the help of professional coaching, because the implementation of the own desired structure is the special challenge of this group. However, inability leads to discomfort. Thus, people of *Eating as self-determination* need to get the feeling of being able to organize eating situations independently, despite the given structure of everyday life.

Due to the adaptability of *The Adaptive* and their well-being with it, it seems debatable whether the introduction of an intervention for this type is necessary. On the one hand, it could be thought that it makes sense to develop own food-related behavior structures in order to minimize the dependency factor on others. On the other hand, this type expresses a good feeling about the nature of their own food-related behavior (40). Accordingly, it could make sense to bring certain family products or community-promoting products onto the market; or to promote the formation of their own structures by means of a systemic approach or a professional coaching.

Special product marketing could be helpful for *Eating as a necessary Evil*. For this type of people, products could be offered on the market that are easy to prepare and at the same time have a high nutrient density or an adapted nutrient profile. Such products should be specially positioned in the supermarket and accompanied by targeted communication measures for this special group (78). Also for *Eating as a way of life*, appropriate products such as vegan, vegetarian, or products that are strongly unprocessed and in harmony with nature should be increasingly brought to the market.

With this study, we have identified and described significant features of the *eating action types* often overlooked and taken a first step toward generalizing the results of the qualitatively built *eating action types*. It can be understood as a preliminary step toward deriving a general typology that does not have a thematic focus, refers to the population as a whole and focuses on the individual. However, the results should be further explored in qualitative and quantitative studies in future in an international context to show whether there are e.g., cultural influences or differences between countries. The results of this study are a valuable tool for nutrition consultancies, food companies, policy makers and advisors to consider the different ways people integrate food into their daily lives in their own specific actions. This may improve the commercial performance of commercial companies, but also increase the effectiveness of policy initiatives and advisory and educational activities in the field of food and eating.

## Data Availability Statement

The raw data supporting the conclusions of this article will be made available by the authors, without undue reservation.

## Ethics Statement

Ethical review and approval was not required for the study on human participants in accordance with the local legislation and institutional requirements. The patients/participants provided their written informed consent to participate in this study.

## Author Contributions

LL conceived, designed, performed the experiments, analyzed the data, and wrote the draft of the paper. AE-K and KM contributed to the writing and evaluation process. All authors contributed to the article and approved the submitted version.

## Funding

The preparation of this paper was supported by the *enable* Cluster and is cataloged by the *enable* Steering Committee as *enable* 05 (http://enable-cluster.de). This work was funded by the German Ministry for Education and Research (BMBF) (Grant number: FKZ01EA1409D). The authors greatly acknowledge this financial support but remain solely responsible for the content of this manuscript.

## Conflict of Interest

The authors declare that the research was conducted in the absence of any commercial or financial relationships that could be construed as a potential conflict of interest.

## Publisher's Note

All claims expressed in this article are solely those of the authors and do not necessarily represent those of their affiliated organizations, or those of the publisher, the editors and the reviewers. Any product that may be evaluated in this article, or claim that may be made by its manufacturer, is not guaranteed or endorsed by the publisher.
